# Experimental Models of Traumatic Injuries: Do They Capture the Coagulopathy and Underlying Endotheliopathy Induced by Human Trauma?

**DOI:** 10.3390/ijms241311174

**Published:** 2023-07-06

**Authors:** Liam Barrett, Nicola Curry, Jeries Abu-Hanna

**Affiliations:** 1 Division of Anaesthesia, Department of Medicine, University of Cambridge, Cambridge CB2 1TN, UK; lb893@cam.ac.uk; 2 Emergency Department, Cambridge University Hospitals NHS Foundation Trust, Cambridge CB2 0QQ, UK; 3 Nuffield Division of Clinical Laboratory Sciences, Radcliffe Department of Medicine, University of Oxford, Oxford OX3 9DU, UK; jeries.abu-hanna@ndcls.ox.ac.uk; 4 Oxford Haemophilia and Thrombosis Centre, Oxford University Hospitals NHS Foundation Trust, Oxford OX3 7LD, UK

**Keywords:** trauma-induced coagulopathy, endotheliopathy of trauma, haemorrhage, coagulation, fibrinolysis, experimental models

## Abstract

Trauma-induced coagulopathy (TIC) is a major cause of morbidity and mortality in patients with traumatic injury. It describes the spectrum of coagulation abnormalities that occur because of the trauma itself and the body’s response to the trauma. These coagulation abnormalities range from hypocoagulability and hyperfibrinolysis, resulting in potentially fatal bleeding, in the early stages of trauma to hypercoagulability, leading to widespread clot formation, in the later stages. Pathological changes in the vascular endothelium and its regulation of haemostasis, a phenomenon known as the endotheliopathy of trauma (EoT), are thought to underlie TIC. Our understanding of EoT and its contribution to TIC remains in its infancy largely due to the scarcity of experimental research. This review discusses the mechanisms employed by the vascular endothelium to regulate haemostasis and their dysregulation following traumatic injury before providing an overview of the available experimental in vitro and in vivo models of trauma and their applicability for the study of the EoT and its contribution to TIC.

## 1. Background

Globally, traumatic injury remains one of the leading causes of death in persons under 44 years of age, accounting for over 4 million deaths every year [1]. This exceeds deaths caused by cardiovascular disease and closely trails behind those caused by infection [2]. Due to an ageing population, traumatic injury is also expected to become a significant cause of morbidity and mortality in older people [3,4]. Uncontrolled haemorrhage accounts for 40% of all trauma-related deaths, and, therefore, controlling haemorrhage and restoring blood volume form the core of the current therapeutic approach to trauma bleeding [5,6,7]. Injury is a global health concern, and the economic burden attributed to the management of patients with trauma haemorrhage is significant; for example, it was estimated that in England the cost of managing haemorrhage after trauma was GBP 168 million per year [8,9]. Despite extensive efforts to improve clinical management and patient outcomes, trauma remains a clinical and socioeconomic issue of major relevance for young people and is becoming a larger issue for the elderly [10].

Impaired coagulation and fibrinolysis following traumatic injury, a clinical phenomenon termed trauma-induced coagulopathy (TIC), have been observed for decades, with temporal changes in coagulation following severe trauma first clinically documented and reported in the 1960s [11,12,13]. TIC denotes a spectrum of clotting abnormalities or phenotypes that range from hypocoagulability and hyperfibrinolysis in the early stages of trauma (“early” TIC; within 6 h of injury), resulting in potentially fatal bleeding, to hypercoagulability and fibrinolysis suppression in the later stages (“late” TIC; 24 h after the injury), leading to multiple organ failure and death [11,12,14,15,16]. Early coagulopathy can develop because of resuscitation with large volumes of cold fluids, which have dilution and hypothermic effects on coagulation factors [16,17]. Therefore, it is important to distinguish between early TIC that occurs because of the trauma itself and the body’s responses to it from iatrogenic trauma-associated coagulopathy [16,17].

**Figure 1:**
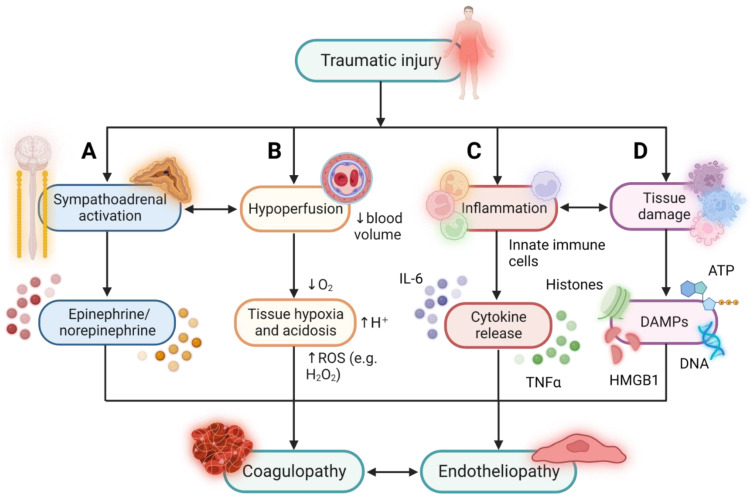
Pathophysiological and immunological responses to traumatic injury converge to induce a dyad of endotheliopathy and coagulopathy. Traumatic injury elicits numerous, complex pathophysiological and immunological responses, including sympathoadrenal activation, tissue damage, inflammation, and tissue hypoperfusion, which are thought to converge to induce systemic endothelial dysfunction (endotheliopathy) and impaired coagulation and fibrinolysis (coagulopathy). (**A**) Hypovolaemic haemorrhage following traumatic injury activates the sympathoadrenal axis, resulting in a surge in the catecholamines epinephrine and norepinephrine. (**B**) Reduced oxygen delivery to tissues due to hypoperfusion results in tissue acidosis and hypoxia, leading to the production of ROS, such as H_2_O_2_, which can further damage cells, including ECs, via protein, lipid, and nucleic acid oxidation. (**C**) Traumatic injury elicits an inflammatory innate immune response, driven primarily by neutrophils and monocytes, that serves to clear damaged tissues of dying cells and to set the stage for tissue healing and repair. (**D**) Tissue damage caused by traumatic injury disrupts macrobarriers, such as the skin and mucosal membranes, and microbarriers, such as cell membranes, resulting in the release of DAMPs, such as nuclear and mitochondrial DNA, histones, ATP, and HMGB1. DAMPs can activate the endothelium directly by signalling via pattern recognition receptors or indirectly by activating innate immune cells and inducing the release of pro-inflammatory cytokines, such as IL-6 and TNFα. This figure was created in BioRender.com. ROS: Reactive oxygen species; H_2_O_2_: hydrogen peroxide; ECs: endothelial cells; DAMPs: damage-associated molecular patterns; ATP: adenosine triphosphate; HMGB1: high-mobility group box 1; IL-6: interleukin-6; TNFα: tumour necrosis factor α.

Traumatic injury is associated with complex pathophysiological and immunological responses, as illustrated in Figure 1 [18,19,20]. Haemorrhage due to severe trauma results in hypovolaemia and hypoperfusion, which render tissues hypoxic, lead to metabolic acidosis, and activate the sympathoadrenal system. When compounded by tissue injury, this so-called haemorrhagic shock is capable of triggering TIC. Injurious insults to tissues lead to extensive necrosis, provoking the release of damage-associated molecular patterns (DAMPs), such as histones, HMGB1, polyphosphates (e.g., ATP), and nuclear and mitochondrial DNA, which are capable of engaging cells of the innate immune system by signalling via pattern recognition receptors [21,22,23,24,25]. Physical trauma also triggers an inflammatory response, characterised by a cytokine “breeze” or “storm”, depending on the severity of the injury [26,27]. Tissue damage and inflammation are thought to synergise with haemorrhagic shock to promote early, hypocoagulable TIC and contribute to delayed hypercoaguability, directly and indirectly via the induction of endotheliopathy [28,29].

The endothelium is among the first cellular barriers to respond to traumatic injury [30]. It is also a key regulator of haemostasis and thrombosis [31], serving to maintain the blood fluidity and patency of intact, healthy vessels, maintaining adequate tissue perfusion while preventing excessive blood loss when vessels are injured [31]. The endothelial response to trauma is poorly understood and can be described as a loss of endothelial normalcy culminating in dysfunction, a phenomenon aptly termed the endotheliopathy of trauma (EoT). Evidence suggests that the EoT may be central to the development of TIC, particularly the temporal switch from hypo- to hypercoagulability that occurs after traumatic injury [11,32]. Our understanding of the mechanisms of the EoT and its contribution to TIC remains in its infancy due to the scarcity of basic science research driven by the lack of experimental models.

Consequently, there is a growing need for the development of experimental models, both in vitro and in vivo, to aid in furthering our understanding of the pathological mechanisms that underlie the EoT and its ability to drive TIC. Traumatic injuries are highly heterogeneous, varying in aetiology, severity, anatomical location, and the pathophysiological responses they trigger. Therefore, experimentally modelling the endotheliopathy that occurs following traumatic injuries remains highly challenging. This review seeks to provide an overview of the endothelial regulation of haemostasis and thrombosis in health and following injury before summarising the in vitro and in vivo models currently employed to study the EoT and its contribution to TIC, particularly following extracranial or peripheral traumatic injuries.

## 2. Endothelial Regulation of Haemostasis in Health and Following Traumatic Injury

**Figure 2:**
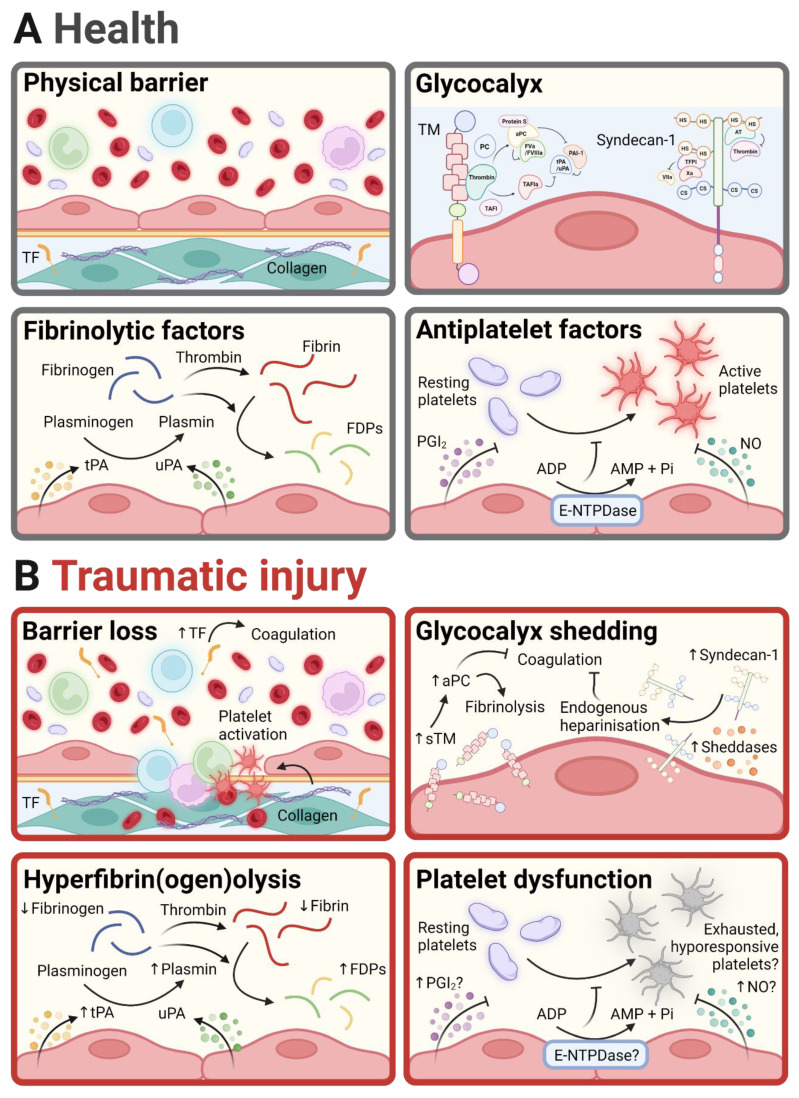
Endothelial regulation of haemostasis and thrombosis in health and following traumatic injury. The endothelium is a key regulator of haemostasis and thrombosis. (**A**) In intact, healthy vessels, the endothelium employs several mechanisms to discourage intravascular coagulation (thrombosis), including the formation of a tight physical barrier with an overlying anticoagulant glycocalyx and the release of fibrinolytic and antiplatelet factors. (**B**) Following traumatic injury, loss of the endothelial barrier leads to the exposure procoagulant and platelet-activating proteins and the extravasation of leukocytes and their ingress into tissues, promoting sterile inflammation. Traumatic injury also results in the shedding of glycocalyx components, resulting in hypocoagulability. When traumatised, ECs increase the release of fibrinolytic factors to promote fibrin(ogen)olysis. Although not yet proven, following traumatic injury, the initial EC hyperactivation may increase the production of platelet inhibitory molecules to confer agonist hyposensitivity upon the platelets. This figure was created in BioRender.com. TF: Tissue factor; TM: thrombomodulin; PC: protein C; aPC: activated PC; TAFI: thrombin-activatable fibrinolysis inhibitor; TAFIa: activated TAFI; FVa: activated factor V; FVIII: activated factor VIII; AT: antithrombin; TFPI: tissue factor pathway inhibitor; FVIIa: activated factor VII; HS: heparan sulphate; CS: chondroitin sulphate; tPA: tissue plasminogen activator; uPA: urokinase-type plasminogen activator; FDP: fibrin degradation product; PGI_2_: prostacyclin; NO: nitric oxide; ADP: adenosine diphosphate; AMP: adenosine monophosphate; E-NTPDase: ecto-nucleoside triphosphate diphosphohydrolase; sTM: soluble thrombomodulin.

Vascular endothelial cells (ECs) constitute the innermost layer of blood vessels and regulate various processes, including the trafficking of leukocytes into tissues, maintenance of selective vascular permeability, inflammation, vascular tone, haemostasis, and thrombosis [31,33]. In intact, healthy vessels, ECs provide an antithrombotic surface that discourages platelet aggregation and fibrin clot formation via the cell surface’s expression and secretion of antiplatelet and anticoagulant molecules (Figure 2), maintaining blood fluidity, vessel patency, and tissue perfusion [33]. Upon vascular injury, normally functioning ECs not only promote the formation of stable, fibrinolysis-resistant clots to halt bleeding but also limit clot formation to regions where vascular integrity needs to be restored [34]. Following traumatic injury, however, ECs become dysfunctional or “traumatised”, a consequence of the injury itself as well as the body’s response to the injury, to promote fibrinolysis in the early stages and thrombosis in the later stages [12]. Our understanding of this EC traumatisation (or the EoT) and its underlying mechanisms and contribution to TIC remains poor. This section discusses the mechanisms employed by ECs to regulate haemostasis and thrombosis in health and their dysregulation following traumatic injury.

Physical barrier. Through intercellular adherens and tight junctions, ECs form a barrier that separates the underlying vascular layers and the surrounding tissue from the blood that flows in the vessel lumen [35]. Through this barrier, ECs conceal pro-coagulant and platelet-activating proteins, such as tissue factor (TF) and collagen [36]. Upon traumatic injury, the loss of endothelial barrier integrity and the consequent increase in vascular permeability results in the exposure of subendothelial TF and the activation of the extrinsic pathway of coagulation, leading to the generation of thrombin and the formation of a fibrin clot. When activated or dysfunctional, such as in response to traumatic injury, ECs can also upregulate the expression of TF on their surface and/or activate latent TF that is constitutively expressed [37,38]. Endothelial denudation following traumatic injury can also expose subendothelial collagen to the blood, resulting in platelet adhesion and activation via two major receptors, integrin α2β1 and glycoprotein (GP) VI [39]. Under conditions of high shear, the GPIb-V-IX receptor complex on platelets and its ligand von Willebrand factor (vWF), which is released from the Weibel–Palade bodies of activated ECs, are additionally required for platelet capture and firmer adhesion.

Glycocalyx. The endothelial glycocalyx is a negatively charged, carbohydrate-rich layer that covers the luminal surface of the vascular endothelium [40,41]. It is composed predominantly of the carbohydrate portions of proteoglycans and glycoproteins, projecting from the surface of the endothelium into the vessel lumen [42]. The endothelial glycocalyx is a dynamic structure for which composition is in a state of constant flux with components removed by the flowing blood (and the shear stress that it exerts) and replenished by the endothelium [43]. It performs a myriad of physiological functions, including the maintenance of a selectively permeable barrier, discouragement of leukocyte adhesion and subsequent extravasation, and prevention of intravascular coagulation.

Among the EC-membrane-spanning proteoglycans is syndecan-1, for which heparan sulphate (HS) side chains inhibit coagulation by interacting with anti-thrombin (AT), which, as its name implies, inactivates thrombin as well as the coagulation factor Xa, and tissue factor pathway inhibitor (TFPI), which inactivates factor VIIa upstream of thrombin generation in the extrinsic pathway of coagulation [44]. Increased plasma levels of syndecan-1 in trauma patients suggest that, following traumatic injury, the degradation of the endothelial glycocalyx and the ensuing release of syndecan-1 with its anti-coagulant HS chains into the blood induces endogenous heparinisation, contributing to the early hypocoagulable phase of TIC [45,46]. It is widely assumed that shed syndecan-1 is a marker of endothelial damage. However, syndecan-1 expression is not restricted to the endothelium with syndecan-1 also present on epithelial cells lining the lungs and intestines [47,48,49].

The glycoprotein thrombomodulin (TM) is also a major constituent of the endothelial glycocalyx. TM spans the EC membrane, and its luminal portion can be shed into the bloodstream as soluble TM (sTM), which retains biological activity [50]. TM has multiple functions owing to its multidomain structure, which allows interactions with several proteins, including thrombin [51,52]. Once bound to TM, thrombin loses specificity for its classical substrate fibrinogen and gains preference for the alternate substrates protein C (PC) and thrombin-activatable fibrinolysis inhibitor (TAFI), generating activated PC (aPC) and activated TAFI (TAFIa). aPC cleaves and inactivates coagulation factors Va and VIIIa, which are cofactors for factors Xa and IXa, respectively, upstream of the conversion of prothrombin to thrombin in the coagulation cascade. aPC also derepresses fibrinolysis via its interaction with and consequent inhibition of PAI-1. TAFIa removes C-terminal lysine residues from fibrin strands as they are being degraded by plasmin so that they can no longer recruit and activate tPA, which would otherwise lead to the further activation of plasminogen to plasmin in a positive feedback loop [53,54].

Elevated levels of sTM have consistently been reported in patients with traumatic injuries and are suggestive of increased TM cleavage and shedding on the endothelial surface [55]. Accompanying elevated sTM levels are increased levels of aPC, which, by inhibiting the assembly of the prothrombinase complex (factor Xa complexed with factor Va) and, therefore, thrombin generation, contribute to the early hypocoagulability in TIC [55,56,57,58]. By inhibiting plasminogen activator inhibitor-1 (PAI-1), aPC also relieves the break on fibrinolysis, leading to increased fibrinogen and fibrin breakdown and contributing to the hyperfibrinolysis seen in trauma patients [55]. The enzymes or sheddases responsible for this endothelial TM cleavage remain largely unknown; however, there is evidence supporting a role for neutrophil-derived proteases. Although universally regarded as a marker of endothelial injury, sTM is not shed exclusively from endothelial surfaces with evidence supporting possible shedding from circulating immune cells, including abundant monocytes, which are also activated by trauma [59,60].

Fibrinolytic factors. ECs produce, store, and release tissue plasminogen activator (tPA) and urokinase-type plasminogen activator (uPA) [61]. Following its synthesis, tPA is stored in subcellular compartments that are distinct from endothelial Weibel–Palade bodies and is released both constitutively and upon EC activation [61]. Following its release, tPA is retained on the endothelial surface, where its activity is amplified by its interaction with membrane receptors, including annexin A2 and S100A10, to generate plasmin from plasminogen [62,63,64,65,66]. Plasmin degrades fibrin strands into fibrin degradation products, restricting fibrin formation or disintegrating an already formed fibrin clot. Plasmin also catalyses the degradation of fibrinogen, reducing its availability for fibrin clot formation [67,68]. ECs also synthesise PAI-1, which opposes the action of tPA on plasminogen and is upregulated following EC activation [69,70]. High levels of circulating tPA in severely injured trauma patients suggest that ECs respond to traumatic injury by releasing copious amounts of tPA [71,72]. Moreover, the increased levels of S100A1 in the plasma of patients with severe traumatic injuries and an occult hyperfibrinolytic phenotype, suggest that, in response to traumatic injury, ECs not only overwhelmingly release tPA but also alter their surface to promote tPA retention and activity [73].

Antiplatelet factors. In addition to discouraging fibrin formation, the endothelium potently inhibits platelet activation and subsequent aggregation by the release of anti-platelet factors, namely, the arachidonic acid metabolite prostacyclin (PGI_2_) and the soluble gas nitric oxide (NO) [74,75]. PGI_2_ passively diffuses across the endothelial membrane into the blood, where it inhibits platelet activation by ligating the Gs-coupled IP prostanoid receptor, resulting in the cytosolic generation of inhibitory cAMP [74]. The endothelial production of PGI_2_ can be augmented by a plethora of agonists, including histamine, bradykinin, and thrombin [76,77]. Although experimental evidence has yet to be provided, it is plausible that endothelial hyperactivation following traumatic injury leads to the overproduction of PGI_2_, which renders platelets hyporesponsive to agonist stimulation.

NO is generated from the amino acid L-arginine by endothelial NO synthase (eNOS) and, similarly to PGI_2_, diffuses across the endothelial membrane to inhibit platelet activation via the stimulation of soluble guanylate cyclase and the generation of inhibitory cGMP within platelets [78]. Endothelial NO generation could be augmented following traumatic injuries, particularly in response to the surge in epinephrine, which is known to induce stimulatory phosphorylation and, as a result, increase the activity of eNOS [79]. ECs also present a catalytically active surface by the expression of the ADP-hydrolysing enzyme, called ecto-nucleoside triphosphate diphosphohydrolase (E-NTPDase) [80]. E-NTPDase hydrolyses ADP to AMP to prevent the purinergic activation of platelets [80]. Whether endothelial E-NTPDase expression and/or enzymatic activity are affected by trauma has yet to be elucidated.

Altered platelet–EC interactions have been proposed to occur after trauma and to contribute to the development of TIC. For example, rapid and sustained increases in the plasma levels of vWF, particularly that of the ultra-large, multimeric form (UL-vWF), together with decreases in plasma levels of the UL-vWF-degrading metalloproteinase ADAMTS13 have been demonstrated in trauma patients, suggesting an augmented release of vWF multimers from the Weibel–Palade bodies of activated ECs immediately following traumatic injury and impaired clearance by ADAMTS13 [81,82]. Increased UL-vWF availability promotes platelet adhesion to subendothelial collagen, which is exposed upon endothelial denudation. Our understanding of the contribution of the EoT to impaired platelet function in trauma patients remains unexplored and warrants urgent investigation.

## 3. In Vitro Models of Traumatic Injury

In vitro models of trauma are helping to dissect the cellular and molecular mechanisms of the EoT and its contribution to TIC. They provide an invaluable platform for performing well-controlled, reproducible experiments and are particularly useful for identifying the stimuli cascades that trigger the induction of the EoT following injury. They can be used to identify therapeutic targets for normalising the traumatised endothelium and its regulation of coagulation and fibrinolysis with their utility determined by their fidelity to in vivo trauma pathobiology. This section discusses the various approaches that have been used to model aspects of the complex pathophysiology of trauma and induce the EoT in vitro. Due to the paucity of in vitro models of the EoT, this section also discusses the applicability of established in vitro models of bleeding for the study of the EoT and its influence on haemostatic processes.

Endothelial models of isolated haemorrhagic shock. Haemorrhagic shock has been simulated in vitro to study its effects on the endothelium. Human umbilical vein ECs (HUVECs), for example, have been subjected to clinically relevant concentrations of epinephrine and norepinephrine, hypoxia followed by reoxygenation, and sublethal concentrations of H_2_O_2_ [83]. Individually, epinephrine and norepinephrine increased sydecan-1 shedding, a marker of glycocalyx damage, with norepinephrine causing significantly greater glycocalyx damage [83]. This syndecan-1 shedding was further augmented by hypoxia/reoxygenation, indicating a possible synergy between sympathoadrenal activation and hypoperfusion in inducing the EoT. TM was also shed in response to treatment with epinephrine or norepinephrine, an effect heightened by hypoxia/reoxygenation and H_2_O_2_ [83]. Norepinephrine, but not epinephrine, stimulated the release of the fibrinolysis mediator tPA from HUVECs, which was magnified by hypoxia/reoxygenation and H_2_O_2_. These data suggest that this in vitro model could be used to study endothelial changes induced solely by haemorrhagic shock under static conditions. This model, however, fails to consider the effects of the flowing blood and the shear stress that it exerts on the luminal endothelial surface, which is known to be a key determinant of the composition of the glycocalyx.

Endothelialised microfluidic models of isolated haemorrhagic shock. Endothelialised microfluidic channels have been used to assess the endothelial dysfunction induced by haemorrhagic shock under blood-flow-mimicking conditions [84]. HUVECs subjected to epinephrine and hypoxia/reoxygenation under flow conditions showed a marked reduction in glycocalyx thickness, a consequence of the increased shedding of the glycocalyx components syndecan-1 and hyaluronic acid into the perfusate [84]. In combination, epinephrine and hypoxia/reoxygenation also induced profibrinolytic tPA secretion by HUVECs, while inhibiting the production of antifibrinolytic PAI-1 [84]. By incorporating the dynamicity of flow, this model mimicked the physiological environment to which ECs are subjected to in vivo and was able to reproduce the endothelial changes that are thought to underlie the hyperfibrinolysis and hypocoagulability in early TIC.

In vitro models utilising patient-derived or traumatised ECs. Currently, there is a lack of in vitro models that fully and accurately recapitulate the complex interplay between the pathophysiological (sympathoadrenal activation and tissue hypoperfusion) and immunological (innate immune responses and surges in pro-inflammatory cytokines) processes that are thought to induce the EoT in injured patients. It is possible that using ECs derived from patients, particularly from their injured or damaged tissues, may circumvent this issue but only if the traumatised phenotype of patient ECs persists in culture or the memory of trauma is reinforced by in vitro stimulation with trauma-related factors, such as catecholamines, hypoxia, pro-inflammatory cytokines, or DAMPs. Obtaining ECs from injured tissues may prove difficult. Alternatively, circulating ECs, which are dislodged from the vessel wall by vascular injury, such as that which occurs in trauma, or endothelial progenitor cells, which are mobilised from the bone marrow by eNOS activation in response to injury and give rise to mature ECs, can be readily obtained from patient blood samples [85,86,87,88,89]. To date, these EC surrogates have not been studied in the context of traumatic injury.

Endothelialised microfluidic models of bleeding. Thus far, two endothelialised microfluidic models of mechanical injury haemorrhage have been described that could potentially be modified and used to model EoT. The device, developed by Sakurai et al., is composed of two channels: an endothelialised microchannel (recapitulating the microvasculature) that opens into a separate “bleeding” microchannel (serving as a bleeding wound) [90]. A pneumatic valve separates the two channels and, when pulled open by negative pressure, mechanically disrupts and, therefore, “injures” the endothelium [90]. Following endothelial “injury”, recalcified whole blood is perfused while the valve is maintained in the open position, allowing it to flow through the endothelialised microchannel as well as the bleeding microchannel [90]. Haemostatic plug formation can be visualised by the fluorescent staining and imaging of platelets and fibrin, and bleeding time (time to cessation of blood flow into the “bleeding” channel after haemostasis) can be determined [90]. The EoT can be incorporated into this model using trauma-patient-derived ECs, as described above, or the treatment of ECs lining the microvascular channel with trauma-related factors.

Another microfluidic model mimicking penetrating vascular injury and the bleeding that ensues was developed by Poventud-Fuentes et al. [91] They introduced a novel microfabricated device, consisting of three interconnected, parallel microchambers, to emulate the compartmentalisation of vascular tissue [91]. The middle chamber or microchannel houses a three-dimensional hydrogel rich in collagen and TF to model the deformable, procoagulant wall of a blood vessel (“vessel wall” chamber) [91]. Along the right side of the middle chamber is an endothelialised microchannel that represents the endothelium-lined lumen of a blood vessel (“intravascular” microchannel) [91]. The microchannel along the left side of the hydrogel-filled middle chamber serves as an extravascular compartment (“extravascular” microchannel) into which blood can escape when the vessel wall is pierced by a microneedle [91]. To assess haemostatic plug formation, the intravascular microchannel is perfused with recalcified blood at a venous shear stress of 100 s^−1^ while fluid is pulled through the extravascular microchannel at a reduced shear of 0.5 s^−1^ to create a transmural pressure gradient across the vessel wall chamber [91]. This pressure gradient forces blood within the intravascular compartment to escape into the extravascular channel upon vessel wall puncture with the microneedle, encountering on its way the procoagulant, collagen/TF-rich hydrogel [91]. The real-time imaging of fluorescently labelled platelets and fibrin allows the visualisation of haemostatic plug formation as well as the quantification of its kinetics [91].

Vascularised organ-on-a-chip. ECs within the vasculature communicate with other cell types, including circulating immune cells within the vessel lumen and stromal (e.g., smooth muscle cells, pericytes, and fibroblasts) and parenchymal cells resident within the surrounding tissue, the behaviour of which is also likely to be influenced by traumatic injuries [92]. In vitro models using EC monocultures exclude these complex cellular interactions that produce overall tissue and organ function. Vascularised organoids (reviewed by Shirure et al. [92]) are better at capturing the multicellular, three-dimensional nature of in vivo human tissues, including stroma, and may be used as in vitro microphysiological systems for the study of endothelial dysfunction in the context of traumatically damaged tissues or organs. The challenge may lie, however, in creating vessels that are large enough to be perfused with whole blood.

## 4. In Vivo Models of Traumatic Injury

Numerous attempts have been made to develop animal models of trauma that reflect the clinical scenario in traumatically injured patients [93,94]. These animal models have been invaluable experimental tools for investigating the pathological mechanisms of traumatic injuries and the preclinical testing of potential intervention strategies [93,94]. Small animals, namely, mice and rats, are most frequently employed in trauma research due to their reasonable cost and ease of handling and genetic manipulation [94]. Furthermore, mice and rats share a considerable proportion (~80%) of their genes with humans [95]. Despite this genetic similarity, however, there remain significant anatomical and physiological differences that limit the ability of rodent models to closely mimic the human response to traumatic injury [96,97]. Larger animals, primarily pigs, have also been heavily used as their anatomy and physiology better resemble those of humans [94]. This section provides an overview of the numerous, diverse models currently used in trauma research, focusing on those that show signs of the EoT and/or non-iatrogenic TIC (Table 1).

Models of isolated trauma. Noble and Collip were the first to develop an in vivo model of isolated blunt trauma, which mimics lethal traumatic injury by causing extensive tissue injury in the absence of gross haemorrhage. To induce blunt trauma, a rotating plastic wheel with internal shelves (the Noble-Collip drum) was used to repeatedly strike down the animal. “Noble-Collip” trauma in rats rapidly induced fibrinolysis activation and subsequent suppression. This was implied by the upregulation of tPA and PAI-1 in various organs, including the kidneys, lungs, and liver, and the increase in plasma levels of inactive tPA-PAI-1 complexes [103]. These findings suggest that blunt trauma results in systemic endothelial dysfunction, a notion further supported by a different study, in which Noble-Collip trauma in rats elevated plasma levels of sTM, which is predominantly shed from the endothelial surface, as is seen in human trauma [104]. In contrast to humans, however, the rise in sTM following injury was not accompanied by increased PC activation, underpinning a possible divergence in the endothelial regulation of haemostasis between humans and rats. In addition, rat models of Noble-Collip blunt trauma showed signs of hypercoagulability, including spontaneous thrombin burst in non-stimulated thrombin generation assays, increased soluble fibrin levels and consumption of platelets, coagulation factors, and antithrombin [104].

Models of isolated haemorrhage. Haemorrhagic shock has been experimentally produced in animals by either fixed-volume, pressure-controlled, or uncontrolled haemorrhage [116]. In models of fixed-volume haemorrhage, a percentage of the total blood volume is withdrawn from the animal over a fixed period in an attempt to recreate the clinical scenario, in which over 40% of circulating blood volume is shed (class IV haemorrhage [117]), resulting in a mortality rate of more than 30% [116]. This model has been used for the assessment of the physiological responses to a single bleeding event and the efficacy of different therapeutic interventions. Coagulation changes, akin to human TIC, however, have not been observed in this model [118]. Furthermore, whether fixed-volume haemorrhage results in endothelial changes resembling human EoT remains unknown. It is, therefore, apparent that models of fixed-volume haemorrhage may not be suited for the study of TIC and the endotheliopathy thought to underlie it.

Models of pressure-controlled haemorrhage involve bleeding the animal until the mean arterial pressure (MAP) drops to a defined level (varying between 29 and 55 mmHg), which is maintained for a set period by repeated haemorrhaging or fluid infusion [116]. It has been argued that this model is superior to that of fixed-volume haemorrhage as the MAP rather than the volume of shed blood can be documented clinically. Although technically challenging, this model has proven an excellent resource for the study of the pathophysiological responses to trauma bleeding. Pressure-controlled haemorrhage has been shown to downregulate the global mRNA expression of sydecan-1 in murine lung, suggesting that, in isolation, haemorrhage can induce some degree of endothelial dysfunction [100].

Animal models of uncontrolled haemorrhages are preferred to models of fixed-volume and pressure-controlled haemorrhage as they more closely resemble the clinical situation of trauma patients with uncontrolled bleeding and allow haemostatic processes to influence the progression of the haemorrhage. Uncontrolled haemorrhage is induced by vascular trauma, including solid organ injury (e.g., splenic injury [102,119] and liver laceration [102,120]), major artery injury (e.g., aortotomy [121], iliac artery tear [122], and femoral artery transection [123]), and appendage amputation (e.g., of the tail [102]). Coagulation variables remained unchanged following uncontrolled haemorrhage in four different rat models, providing strong evidence that haemorrhage alone may be insufficient to induce TIC [102]. Moreover, coagulation parameters were unaltered in porcine models of hepatic-injury-induced uncontrolled haemorrhage [124], also suggesting that sole haemorrhage is not a driver of TIC.

Models of combined haemorrhage and trauma. In models of combined haemorrhage and trauma, animals are subjected to haemorrhage as well as a single traumatic-injury-causing event. Mice subjected to a combination of pressure-controlled haemorrhage and laparotomy showed marked elevations in activated partial thromboplastin time (aPTT) and levels of aPC, mimicking clinical laboratory observations in trauma patients and demonstrating aPC-driven acute coagulopathy [105]. However, individually, pressure-controlled haemorrhage and laparotomy failed to induce these coagulopathic changes, suggesting synergy is required between haemorrhage and trauma to elicit acute coagulopathy [105]. Interestingly, blockading aPC via the administration of a monoclonal antibody normalised aPTT [105]. In a similar murine model, Barry et al. demonstrated increased vascular permeability due to the inflammation-driven loss of interendothelial junctional proteins [106]. Rats subjected to pressure-controlled haemorrhage and laparotomy showed coagulopathic changes as well as increases in the plasma levels of PAI-1, tPA, and syndecan-1, which are indicative of a traumatised endothelium.

Models of combined haemorrhage and polytrauma. Animal models of combined haemorrhage and polytrauma have been shown to better recapitulate severe human trauma, defined by an anatomical injury severity score >15, and to consistently show signs of coagulopathy and endotheliopathy. For example, Wallen et al. used a murine model of polytrauma, in which mice were subjected, in sequence, to weight-drop-induced traumatic brain injury, pressure-controlled haemorrhage with resuscitation, and laparotomy with bilateral abdominal rectus muscle crush injury to induce the EoT [109]. Although sydecan-1 levels were elevated in response to isolated haemorrhagic shock and the combination of shock and polytrauma, plasma levels of sTM increased only when shock was compounded by polytrauma [109]. These data suggest that haemorrhage alone induces a single aspect of the EoT, which is syndecan-1 shedding, whereas both haemorrhage and trauma synergise to cause the cleavage and shedding of both syndecan-1 and TM. Furthermore, pressure-controlled haemorrhage and polytrauma induced coagulopathic changes in mice that were abrogated by the knock-in of a mutant TM gene incapable of activating PC [56].

Similarly, in rat models of combined haemorrhage and polytrauma, coagulation changes, including the prolongation of PT and aPTT, decreased fibrinogen levels, and impaired clot stability [110], were also reported and found to associate with inflammatory responses [125]. Underlying these coagulation changes were increases in plasma tPA and plasmin levels shortly followed by increases in plasma PAI-1 levels, suggesting the activation and subsequent suppression of the fibrinolytic system [111]. Plasma levels of sTM also increased following the combined haemorrhage and polytrauma with no increase, however, in plasma levels of aPC [111]. These findings suggest that animals must be subjected to shock, injury, and tissue damage to induce full-blown EoT and consequent TIC.

## 5. Conclusions

Despite many models being described, there appears to be a significant lack of in vitro and in vivo models of peripheral trauma that can be used to further our understanding of the contribution of the EoT to TIC. With respect to modelling trauma in vitro, studies have thus far only assessed the effect of haemorrhagic shock, which constitutes one aspect of trauma pathology and may not be sufficient to alter the haemostatic function of ECs in patients to give rise to TIC. Using ECs or their surrogates, such as circulating ECs or bone-marrow-derived endothelial progenitor cells, obtained from patients with traumatic injuries may prove superior as they would possess the traumatised phenotype of the patients or memory of it, which can be reinforced by in vitro stimulation with trauma-related factors. Moreover, in vitro models have yet to examine the interaction of ECs with the coagulation and fibrinolysis systems and the impact of haemorrhagic shock and other aspects of trauma pathology, which can be performed using established haemostatic assays, including fluorometric thrombin generation and turbidimetric clot lysis assays, in the presence of cultured ECs. Better yet, the endotheliopathy induced by trauma could be modelled using in vitro microfluidic systems that more closely resemble the physiological environment that ECs are subjected to in vivo and enable the integration of the biologically active, deformable 3D extravascular tissue and its components, the effects of which are often neglected. In such dynamic in vitro systems, faithful to the complexity of the vasculature, haemostatic plug formation, structure, stability, and lysis could be visualised and monitored using fluorescent staining and imaging of platelets and fibrin(ogen) within whole blood following the simulation of mechanical vascular injury. Future in vitro studies must also consider the heterogeneity of the endothelium and its implications, as, with the advent of single-cell omics, ECs within different vascular beds have been shown to exhibit distinct differences in phenotype and function and may, therefore, respond differently to trauma [126,127].

With regard to in vivo models, although a plethora of models have been developed and described, they have not been very well characterised in terms of the coagulopathic and associated endothelial changes. This is likely attributable to our poor understanding of the endotheliopathy and coagulopathy that occur in traumatised patients. Overall, in vivo evidence suggests that haemorrhage alone, whether controlled or uncontrolled, might not be sufficient to induce coagulopathy and the associated endotheliopathy. However, when compounded by trauma, including bone fractures, solid organ damage, and soft tissue injury, haemorrhage is more likely to result in coagulopathic and endothelial changes comparable to those observed in trauma patients. Furthermore, to date, in vivo investigations have been limited to understanding the acute or early phase of TIC, in which the processes of hypocoagulability and hyperfibrinolysis tend to predominate. Long-term trauma models that simulate the clinical trajectory of trauma and, therefore, can be used to assess the temporal changes in the phenotype of ECs and their regulation of coagulation and fibrinolysis are missing. There are also no in vivo models that recapitulate the clinical scenario in which patients survive the trauma and haemorrhage but go on to develop late TIC, which is characterised by hypercoagulability and multiple organ failure.

## Figures and Tables

**Table 1:** Coagulopathic and endothelial changes in various in vivo models of trauma.

Induction of Haemorrhage and/or Trauma	Species	Coagulopathic Changes	Endothelial Changes	Reference
**Models of isolated haemorrhage**				
**Haemorrhage:** fixed-volume (35% of total blood volume)	Pig	↔ PT, ↔ aPTT, ↓ fibrinogen, ↑ fibrinogen degradation	-	Martini et al. [98]
**Haemorrhage:** fixed-volume (20% of total blood volume)	Rat	↔ PT, ↔ aPTT, ↓ fibrinogen, ↑ factor II, ↔ factor V, ↑ factor X	-	Gangloff et al. [99]
**Haemorrhage:** pressure-controlled (35 mmHg for 90 min)	Mouse	-	↓ syndecan-1 mRNA in lungs	Chipman et al. [100]
**Haemorrhage:** pressure-controlled (35 mmHg for 90 min)	Mouse	-	↓ syndecan-1 mRNA in lungs	Wu et al. [101]
**Haemorrhage:** uncontrolled (tail amputation, liver punch biopsy, liver laceration, and spleen transection)	Rat	↔ PT, ↔ aPTT, ↔ fibrinogen, ↔ FDPs, ↔ platelets	-	Morgan et al. [102]
**Models of isolated trauma**				
**Trauma:** Noble–Collip drum-induced blunt trauma	Rat	-	↑ sTM, ↔ aPC, ↑ tPA, ↑ PAI-1, ↑ tPA-PAI-1 complex	Hayakawa et al. [103]
**Trauma:** Noble–Collip drum-induced blunt trauma	Rat	↑ thrombin, ↑ soluble fibrin, ↓ platelets, ↓ antithrombin	-	Hayakawa et al. [104]
**Trauma:** closed mid-shaft limb fractures, laparotomy, and splenic crush	Rat	↔ PT, ↔ aPTT, ↓ fibrinogen, ↑ factor II, ↔ factor V, ↑ factor X	-	Gangloff et al. [99]
**Models of combined haemorrhage and trauma**				
**Haemorrhage:** pressure-controlled (35 mmHg for 60 min) **Trauma:** laparotomy	Mouse	↑ aPTT	↑ aPC	Chesebro et al. [105]
**Haemorrhage:** pressure-controlled (35 mmHg for 90 min) **Trauma:** laparotomy	Mouse	-	↓ VE-cadherin, ↓ ZO-1	Barry et al. [106]
**Haemorrhage:** pressure-controlled (30 mmHg) **Trauma:** unilateral femur fracture with soft tissue damage	Pig	↔ PT, ↔ aPTT, ↓ fibrinogen	-	White et al. [107]
**Haemorrhage:** pressure-controlled (35–40 mmHg for 60 min) **Trauma:** laparotomy	Rat	↑ PT, ↑ aPTT, ↑ D-dimer, ↑ plasmin–antiplasmin complex	↑ PAI-1, ↑ tPA, ↑ syndecan-1	Xu et al. [108]
**Models of combined haemorrhage and polytrauma**				
**Haemorrhage:** pressure-controlled (30 mmHg for 90 min) **Trauma:** weight-drop-induced traumatic brain injury and laparotomy with bilateral abdominal rectus muscle crush injury	Mouse	-	↑ syndecan-1, ↑ sTM	Wallen et al. [109]
**Haemorrhage:** pressure-controlled (40 mmHg) **Trauma:** right femur fracture and damage to small intestine, right and medial hepatic lobes, and skeletal muscle of right hind limb	Rat	↑ PT, ↑ aPTT, ↓ fibrinogen, ↓ platelets, ↓ clot stability	-	Darlington et al. [110]
**Haemorrhage:** pressure-controlled haemorrhage (40 mmHg) **Trauma:** right femur fracture and damage to small intestines, left and medial hepatic lobes, and skeletal muscle of right hind limb	Rat	↑ plasmin	↑ TM, ↔ aPC, ↑ tPA, ↑ PAI-1	Wu et al. [111]
**Haemorrhage:** fixed-volume (20% of total blood volume) **Trauma:** closed mid-shaft limb fractures, laparotomy, and splenic crush	Rat	↑ PT, ↑ aPTT, ↓ fibrinogen, ↑ factor II, ↔ factor V, ↑ factor X	-	Gangloff et al. [99]
**Haemorrhage:** pressure-controlled (40–50 mmHg) **Trauma:** laparotomy and bilateral mid-shaft closed tibia and fibula fractures	Rat	↓ fibrinogen, ↑ plasmi–antiplasmin complex	↑ syndecan-1, ↑ aPC, ↑ sP-selectin	Xu et al. [112]
**Haemorrhage:** pressure-controlled (40–50 mmHg) **Trauma:** laparotomy and bilateral mid-shaft tibia and fibula fractures	Rat	↑ PT, ↑ aPTT	-	Frith et al. [113]
**Haemorrhage:** pressure-controlled (40 mmHg) **Trauma:** small intestinal crush, liver injury, and right femur fracture	Pig	↑ PT, ↓ fibrinogen, ↓ antithrombin III	-	Duan et al. [114]
**Haemorrhage:** pressure-controlled **Trauma:** blunt chest trauma and liver laceration	Pig	↑ PT, ↓ fibrinogen	-	Mohr et al. [115]
**Haemorrhage:** pressure-controlled (30 mmHg) **Trauma:** right mid-shaft femur fracture and hind limb soft tissue injury	Pig	↔ PT, ↔ aPTT, ↓ fibrinogen	-	White et al. [107]
**Haemorrhage:** pressure-controlled (25–30 mmHg for 60 min) **Trauma:** laparotomy and bilateral mid-shaft tibia and fibula fractures	Mouse	↑ D-dimer, ↓ fibrinogen	↑ aPC	Davenport et al. [56]

↑: Increase; ↓: decrease; ↔: no change; PT: prothrombin time; aPTT: activated partial thromboplastin time; FDPs: fibrin degradation products; sTM: soluble thrombomodulin; tPA: tissue plasminogen activator; PAI-1: plasminogen activator inhibitor-1; VE-cadherin: vascular endothelial-cadherin; ZO-1: zonula occludens-1; aPC: activated protein C; sP-selectin: soluble P-selectin.
